# Mycobacteriophage D29 Lysin B exhibits promising anti-mycobacterial activity against drug-resistant *Mycobacterium tuberculosis*

**DOI:** 10.1128/spectrum.04597-22

**Published:** 2023-10-06

**Authors:** Amit Kumar Singh, Rutuja Gangakhedkar, Hemant Singh Thakur, Sunil Kumar Raman, Shripad A. Patil, Vikas Jain

**Affiliations:** 1 Experimental Animal Facility, ICMR-National JALMA Institute for Leprosy and Other Mycobacterial Diseases, M. Miyazaki Marg, Tajganj, Agra, Uttar Pradesh, India; 2 Microbiology and Molecular Biology Laboratory, Department of Biological Sciences, Indian Institute of Science Education and Research, Bhopal, India; 3 Pharmaceutics and Pharmacokinetics Division, CSIR-Central Drug Research Institute, Lucknow, India; University of Nebraska Medical Center, Omaha, Nebraska, USA

**Keywords:** tuberculosis, antibiotic resistance, D29 mycobacteriophage, mycolyl-arabinogalactan esterase, phage therapy, additive effect

## Abstract

The emergence of antibiotic resistance in *Mycobacterium tuberculosis* has been the culprit behind the inefficient treatment of tuberculosis (TB). Mycobacteriophages being rapid and specific in their activity against mycobacteria including *M. tuberculosis* are an emerging alternative solution to the antibiotic resistance problem. D29 mycobacteriophage is known as an efficient lytic phage. Its lytic cassette encodes for two lytic proteins, Lysin A (LysA) and Lysin B (LysB), and a holin protein responsible for the translocation of the former two. LysB is a mycolyl-arabinogalactan esterase, which has shown a promising lytic effect on *M. tuberculosis*. In this study, we explored the lytic efficiency of D29 mycobacteriophage LysB protein on drug-resistant strains of *M. tuberculosis*. We demonstrate that LysB effectively lyses multi-drug-resistant *M. tuberculosis* both alone and along with anti-TB drugs. We also report that the surfactant Tween-80, although not vital, potentiates LysB activity. We are able to demonstrate that LysB can eliminate *M. tuberculosis* residing in mouse macrophages. Thus, by combining our *in vitro* and *ex vivo* data, we are able to claim that LysB is a potential phage-derived therapeutic which should undergo pre-clinical and clinical trials.

**IMPORTANCE:**

To combat the rapidly emerging drug-resistant *M. tuberculosis*, it is now essential to look for alternative therapeutics. Mycobacteriophages can be considered as efficient therapeutics due to their natural ability to infect and kill mycobacteria including *M. tuberculosis*. Here, we have exploited the mycolyl-arabinogalactan esterase property of LysB encoded from mycobacteriophage D29. This study is novel in terms of targeting a multi-drug-resistant pathogenic strain of *M. tuberculosis* with LysB and also examining the combination of anti-TB drugs and LysB. All the experiments include external administration of LysB. Therefore, the remarkable lytic activity of LysB overcomes the difficulty to enter the complex cell envelope of mycobacteria. Targeting the intracellularly located *M. tuberculosis* by LysB and non-toxicity to macrophages take the process of the development of LysB as a drug one step ahead, and also, the interaction studies with rifampicin and isoniazid will help to form a new treatment regimen against tuberculosis.

## INTRODUCTION

Tuberculosis (TB), despite being one of the oldest diseases, ranks second among the leading infectious killers after COVID-19 and is overall positioned at 13th place among the leading causes of death (1). More precisely, one-fourth of the world’s population is estimated to harbor *Mycobacterium tuberculosis* infection (2). Despite being declared as a global emergency by the World Health Organization in 1993, the disease is far away from elimination (3). Havoc has been created by the emergence of multi-drug-resistant (MDR) and extensively drug resistant (XDR) strains, which are resistant to most of the available drugs. A significant part of health-care activity and research is dedicated to the prevention of the spread of *M. tuberculosis* besides identification and development of novel therapeutics against it. The standard antibiotic regimen for the treatment of tuberculosis consists of daily administration of an anti-TB drug regimen consisting of four drugs, i.e., isoniazid, rifampicin, ethambutol, and pyrazinamide for 2 months followed by isoniazid and rifampicin thrice weekly for 4 months (4). Despite its proven clinical efficacy, longer treatment duration and hepatotoxicity associated with prolonged administration result in treatment dropout and has raised the probability of the emergence of drug-resistant strains (5).

Concerns about bacterial resistance to antibiotics have prompted researchers to develop new ways or to modify old ways to combat the problem, resulting in the exploration of mycobacteriophages (viruses or phages that infect and kill mycobacterial species) and their related enzymes as new therapeutics to fight against TB. Bacteriophages have been proposed for decades for the treatment of common bacterial infections (6). Phages can combat undesirable bacterial growth by infecting and replicating within their bacterial hosts. The ability of phages to increase in number within the host cell, along with their co-functioning with antibiotics, has led to consideration of phage therapy as a possible therapeutic alternative for the treatment of infections caused by MDR *M. tuberculosis* strains (7 – 9). Endolysins or lysins are the phage-encoded lytic enzymes that are capable of degrading cell wall peptidoglycan of host cells to release progeny phages and are the potential anti-bacterial regardless of the drug sensitivity profile of host cells. Phage lysins have demonstrated the efficacy in diminishing growth of antibiotic-resistant microorganisms and have exhibited low likelihood of developing bacterial resistance (10). A study showed that Lysin B (LysB) of mycobacteriophage Ms6 has lipolytic activity and can hydrolyse mycolic acids from the mycolylarabinogalactan–peptidoglycan complex and trehalose-6,6′-dimycolate (TDM), a trehalose diester of two mycolic acid molecules, from the outer membrane of the mycobacterial cell wall (11). Recently, a few studies have addressed the anti-mycobacterial effect of LysB against *Mycobacterium smegmatis* and *M. tuberculosis*. However, previous studies used either D29 mycobacteriophage or LysB from mycobacteriophages other than D29 mycobacteriophage, which has proven its efficacy repeatedly against *M. tuberculosis* (12 – 15). Furthermore, these studies utilized either *M. smegmatis*, a rather distant relative of *M. tuberculosis*, as the surrogate strain which has a different cell wall composition as compared to *M. tuberculosis* or auxotrophic strain of *M. tuberculosis* mc^2^7902, having a different growth profile inside macrophages and lack of lethality in immunocompromised and immunocompetent mice, for the evaluation of anti-mycobacterial efficacy (16 – 19). Moreover, previous studies were limited by use of Tween-80 in the drug susceptibility assays, which is known to alter mycobacterial drug susceptibility and potentiate LysB activity (11, 14, 17). Interestingly, D29 mycobacteriophage-derived LysB can also act externally, suggesting its promising anti-microbial effect (20). However, these studies have not looked into the efficacy of D29 LysB activity against MDR isolates or evaluated its efficacy against mycobacteria residing inside the macrophages.

In this study, we examined the ability of D29 LysB to kill *M. tuberculosis* so as to use it as a therapeutic alternative to antibiotics. Our data show that D29 LysB targets both drug-sensitive and drug-resistant *M. tuberculosis* isolates, irrespective of its resistance to isoniazid and rifampicin and, importantly, is able to inhibit the growth of intracellular mycobacteria. The anti-bacterial activity of the enzyme was measured alone as well as in combination with anti-TB drug rifampicin against the *M. tuberculosis* MDR isolate. Our results, demonstrating the activity of LysB against *M. tuberculosis*, open a new avenue to explore it as a novel tool to eradicate mycobacteria cells and treat TB.

## RESULTS

### Purified LysB of D29 mycobacteriophage is able to kill *M. tuberculosis in vitro*

D29 mycobacteriophage encodes three proteins from its lytic cassette, viz., Lysin A (LysA) (coded by the gene *gp10*), holin (coded by the gene *gp11*), and LysB (coded by the gene *gp12*) (21). Here, we attempted to develop LysB, an esterase involved in disrupting the mycolic acid layer, as a potential “enzybiotic” that is able to kill *M. tuberculosis*.

**FIG 1:**
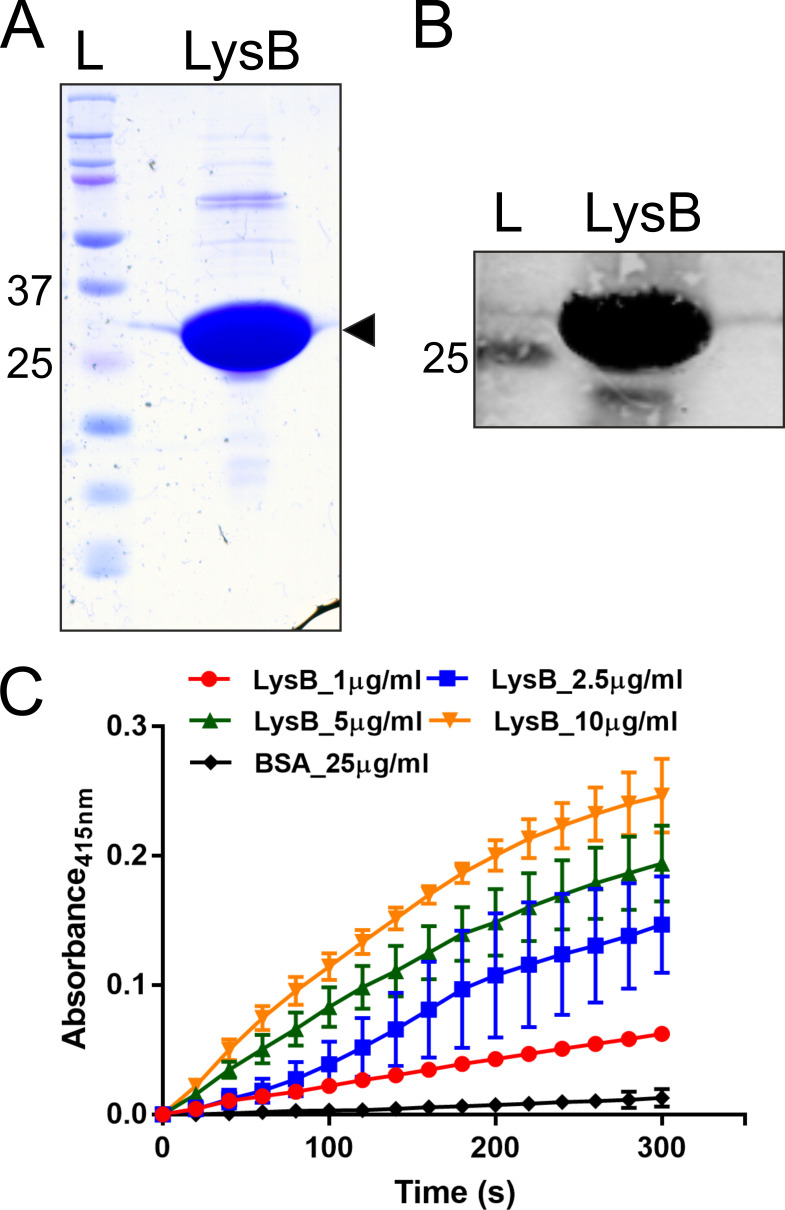
Purified LysB of D29 is an active esterase. Panel A shows a Coomassie Brilliant Blue-stained SDS-polyacrylamide gel with purified D29 LysB (marked with an arrowhead). Panel B shows the Western blot of this protein using anti-LysB antibodies. In both panels, ‘L’ is the protein ladder with molecular weights marked in kilodalton. Panel C shows the activity assay of LysB. The assay was carried out by incubating the substrate nitrophenyl butyrate with purified LysB or BSA, and the reaction was monitored by measuring the absorbance of released 4-nitrophenol at 415 nm with time. Different concentrations of LysB (1.0-, 2.5-, 5.0-, and 10.0-µg/mL reaction volumes as indicated) were taken for the assay. BSA was used as negative control. In this panel, the data represent an average of at least three experiments with error bars representing standard deviation with 99% significant score. BSA, bovine serum albumin.

We cloned, expressed, and purified D29 LysB from *Escherichia coli*. The purified protein was examined on a Coomassie blue-stained polyacrylamide gel (Fig. 1A) and was assessed by Western blotting using the anti-LysB antibodies (Fig. 1B). Next, the protein’s esterase activity was examined *in vitro* using 4-nitrophenyl butyrate as the substrate. Our LysB enzyme assay clearly shows an increase in the absorbance at 415 nm with time, which is due to the release of p-nitrophenol from the substrate demonstrating the action of LysB on it (Fig. 1C). This profile was monitored with increasing concentration of the LysB, along with bovine serum albumin (BSA) acting as a negative control. The data thus confirm that the purified enzyme is active *in vitro*.

**FIG 2:**
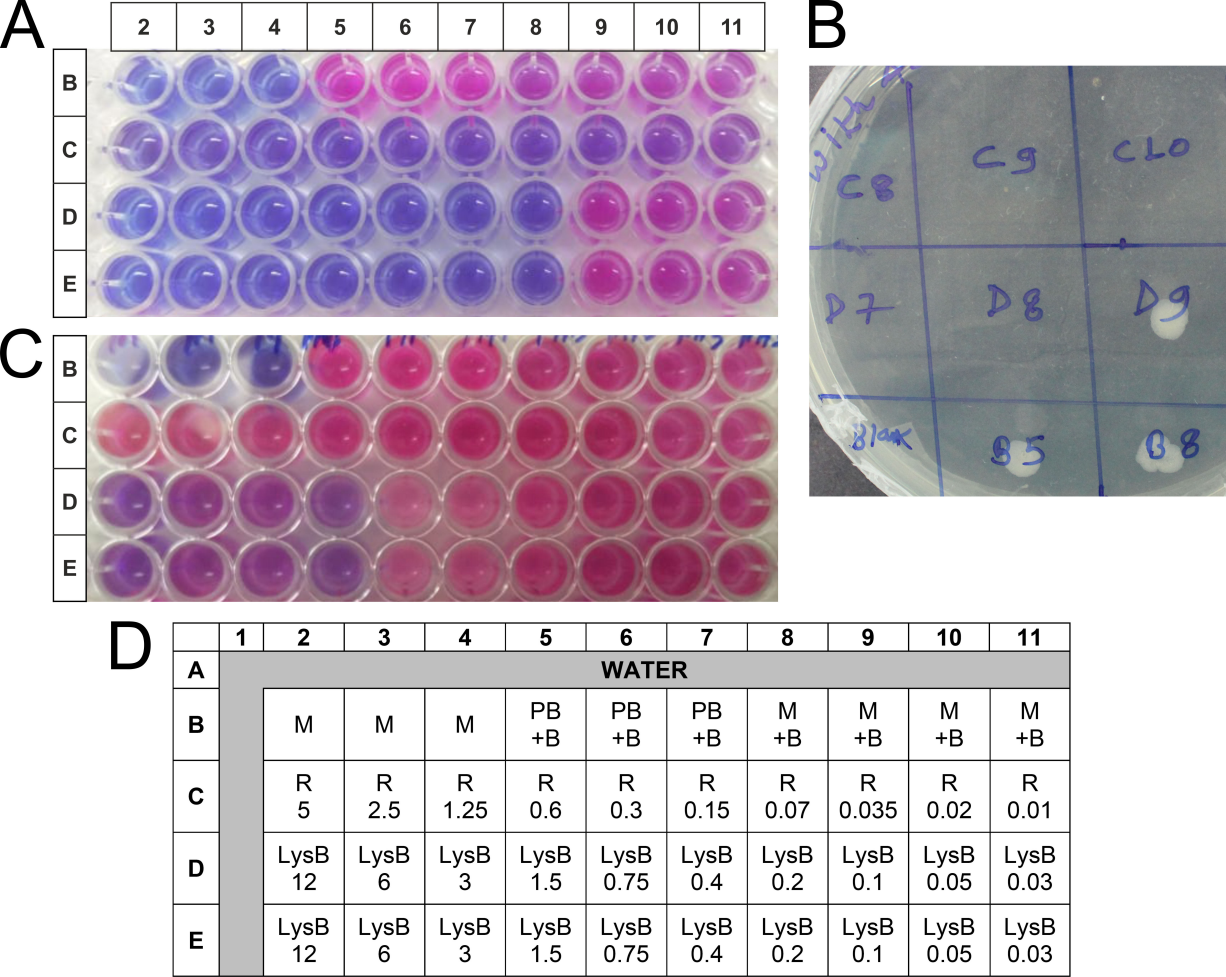
The *in vitro* anti-mycobacterial activity of D29 mycobacteriophage-derived LysB against *M. tuberculosis* was measured using the REMA assay. Mycobacteria were incubated with different concentrations (0.03–12.0 µg/mL) of the LysB against *M. tuberculosis* (A and C, respectively). (**A**) The 1 × 10^4^ CFU/well of *M. tuberculosis* in 100 µL of medium was treated with 100 µL of different concentration of LysB for 6 days. The viability of mycobacteria was determined by a change in color of the resazurin dye; the blue color shows the anti-*M. tuberculosis* activity/effect, and the pink color indicates bacterial growth. (**B**) Culture (3 µL) from selected wells from panel A was spotted on MB 7H11 agar plate to confirm the REMA results. (**C**) *M. tuberculosis* (2 × 10^5^ CFU/well) in 100 µL of medium was treated with 100 µL of different concentrations of LysB for 48 h. The viability of mycobacteria was determined by a change in color of the resazurin dye added on the third day. (**D**) Plate layout plan for the experiment. B, *M. tuberculosis* H37Rv bacteria; LysB, Lysin B; M, 7H9 medium; M + B, *M. tuberculosis* H37Rv growth controls (without any treatment); PB, buffer used for suspending lyophilized protein; R, rifampicin; REMA, resazurin microtiter assay plate.

We next measured minimum inhibitory concentration (MIC) of LysB for *M. tuberculosis* using the microtiter plate-based colorimetric assay. Some studies have assessed the inoculum’s size effect and noted the increase in MIC of an antibiotic with increase in inoculum size, which may increase the probability of emergence of drug resistance (22). We, therefore, investigated the effect of different inoculum sizes (1 × 10^5^ CFU/mL and 2 × 10^6^ CFU/mL) on the susceptibility of *M. tuberculosis* H37Rv to LysB and rifampicin. The MIC of LysB for *M. tuberculosis* following the standard protocol (22) using 10^5^ CFU/mL of mycobacteria was found to be 0.20 µg/mL (Fig. 2A; Table 1). The rifampicin MIC was found to be 0.02 µg/mL and was similar to the MIC reported elsewhere (22). A few microliters of the different samples were tested on the Middlebrook (MB) agar plate for the bacterial growth (Fig. 2B), which further confirmed the killing of *M. tuberculosis*.

**TABLE 1:** MIC for D29 mycobacteriophage-derived LysB and anti-TB drug rifampicin against *M. tuberculosis* at different inoculum concentrations

Organism	Inoculum size (CFU/mL)	D29LysB (µg/mL)	Rifampicin (µg/mL)
***M. tuberculosis*** **H37Rv**	10^5^	0.20	0.02
	10^6^	12.0	>5.0
**Fold change in MIC**		60.0	>250.0

We noticed that an increase in inoculum size affected the activity of both LysB and rifampicin. The increase in MIC value was more prominent for rifampicin (from 0.02 to >5.0 µg/mL), while for LysB, the change was moderate (from 0.2 to 12.0 μg/mL, Fig. 2C; Table 1). The plausible mechanism for change in MIC may be attributed to the increase in bacterial density resulting in reduced ratio of available drug molecules per target rendering them less effective in killing mycobacteria (23).

**FIG 3:**
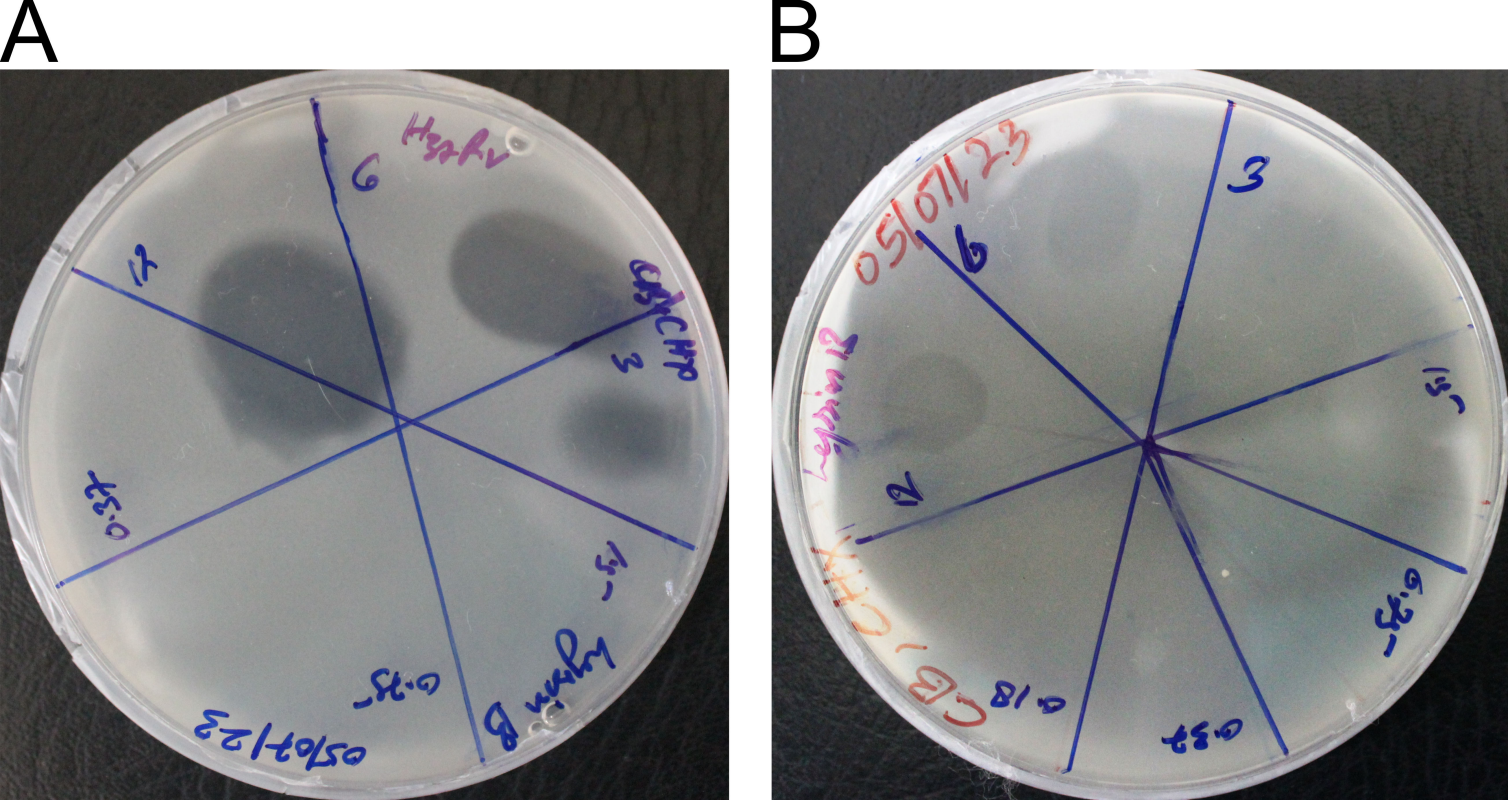
The anti-mycobacterial activity of LysB was determined by plate lysis assay on MB 7H11 medium plate in the (**A**) presence or (**B**) absence of Tween-80. Anti-mycobacterial activity is indicated by the formation of a lytic zone. In the presence of Tween-80, the clear spot zone was observed at 3 µg/mL. In the absence of Tween-80, the concentration up to which a clear spot zone was observed increased and was visible at 6 µg/mL.

We also tested the anti-microbial activity of LysB against *M. tuberculosis* in a plate lysis assay in the presence and absence of Tween-80. The presence of Tween-80 potentiated the LysB activity, and *M. tuberculosis* was susceptible to the lowest protein concentration used (3 µg/mL, Fig. 3A). In the absence of Tween-80, the lowest concentration of LysB resulting in a clear spot zone indicating cell lysis shifted to 6 µg/mL (Fig. 3B). These results demonstrate that LysB is a potent molecule against *M. tuberculosis*, which can function at a very low concentration. D29 LysB enzyme in this study exhibits significant anti-bacterial activity in both presence and the absence of Tween-80. However, the enhanced anti-mycobacterial activity observed in the presence of Tween-80 could be attributed to the action of oleic acid released by hydrolysis of Tween-80 by LysB.

### LysB is an effective anti-bacterial against MDR TB isolate *in vitro* and shows additive effect with anti-TB drug rifampicin

**FIG 4:**
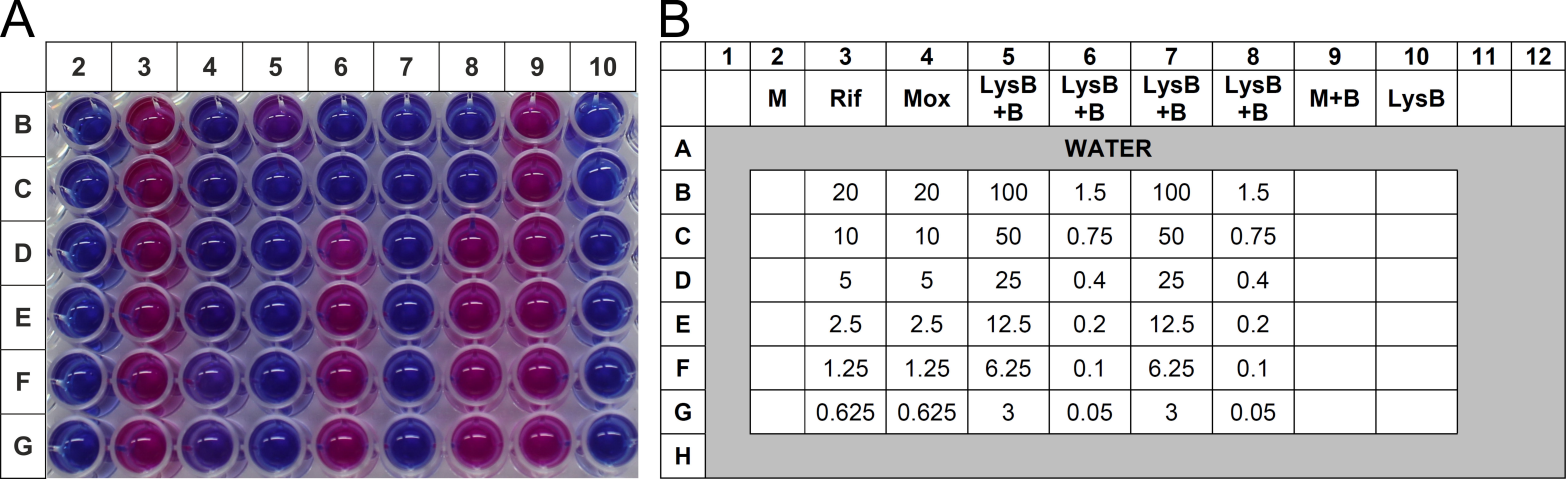
Effect of LysB against *M. tuberculosis* MDR isolate using the REMA assay. (**A**) Mycobacteria were incubated with different concentrations (0.0625–20.0 µg/mL) of the anti-TB drugs rifampicin or moxifloxacin or LysB (concentration ranging from 0.05 to 20.0 µg/mL) for 6 days. The viability of mycobacteria was determined by a change in color of the REMA; the blue color shows the anti-M. *tuberculosis* activity/effect, and the pink color indicates bacterial growth. (**B**) Plate layout of the experiment shown in panel A. B, *M. tuberculosis* bacteria; LysB, well containing only Lysin B protein; M, blank media control without inoculation; M + B, *M. tuberculosis* growth control (without any treatment); Mox, mycobacteria treated with moxifloxacin; Rif, mycobacteria treated with rifampicin.

The *in vitro* anti-mycobacterial activity of LysB was also determined against the previously characterized MDR TB clinical isolate (JAL-19187) procured from the repository of National JALMA Institute for Leprosy and Other Mycobacterial Diseases [Indian Council of Medical Research (ICMR)], Agra, India. Resazurin microtiter assay plate (REMA) was used to determine the MIC values of LysB against the MDR isolates (Fig. 4). LysB was found to be active against the *M. tuberculosis* MDR isolate, with an MIC value of 0.75 µg/mL (Table 2). The clinical isolate of *M. tuberculosis* was resistant to the first-line anti-TB drug rifampicin and was susceptible to the second-line anti-TB drug moxifloxacin (24).

**TABLE 2:** Drug sensitivity profile of *M. tuberculosis* MDR isolate against standard drugs (MIC in μg/mL)

Drug	Observed MIC against *M. tuberculosis* MDR isolate (JAL-19187) (µg/mL)	Critical concentration (µg/mL)
**LysB**	0.75	
**Rifampicin**	>20.0	2 (25)
**Moxifloxacin**	0.625	2 (26)

**FIG 5:**
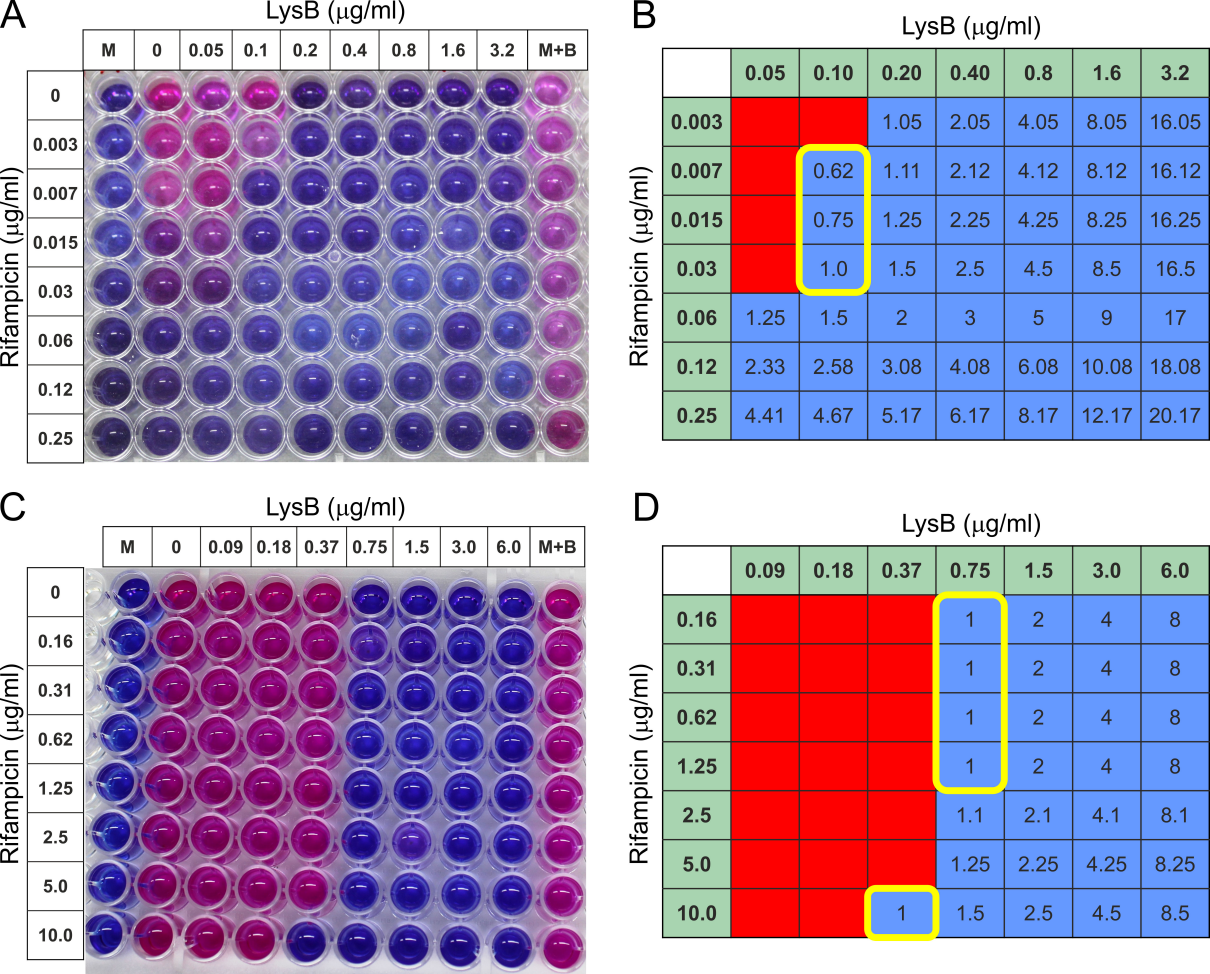
Checkerboard microtiter plate assay testing the combination of rifampicin and LysB against drug-sensitive *M. tuberculosis* H37Rv and multi-drug-resistant *M. tuberculosis* isolate. (**A**) *M. tuberculosis* H37Rv strain: twofold serial dilutions were performed ranging from 0.05 to 3.2 µg/mL for LysB and from 0.003 to 0.25 µg/mL for rifampicin. The addition of resazurin dye revealed the viability of the bacteria. (**C**) *M. tuberculosis* MDR isolate: twofold serial dilutions were performed ranging from 0.1 to 6.0 µg/mL for LysB and from 0.16 to 10.0 µg/mL for rifampicin. The addition of resazurin dye revealed the viability of the bacteria. (B and D) FICs: wells corresponding to no growth by color development (blue color) during REMA assay were shaded blue, while wells corresponding to growth by visual inspection of color (pink color) developed during REMA assay were shaded red. Values for LysB and rifampicin along *x*- and *y*-axes, respectively, represent the concentration of the drug in that column or row alone. The value in each cell is the FICI, or the sum of the FICs, i.e., the ratio of the concentration of the drug in that column or row to the minimum inhibitory concentration of that drug alone of the two drugs in that well. The yellow line-bordered box encloses wells with a FICI of ≤1. The combination is considered additive because the minimum FICI is 0.62 and 1.0 for *M. tuberculosis* drug-susceptible and MDR isolates, respectively. FIC, fractional inhibitory concentration.

Next, we evaluated the impact of LysB in combination with anti-TB drug rifampicin on *M. tuberculosis* H37Rv (drug-sensitive strain) and MDR isolate, respectively, using the checkerboard assay. The fractional inhibitory concentration (FIC) for each combination was calculated and is presented in Table 3 and in Fig. 5A through D. In checkerboard titrations, LysB displayed additive activity against drug-sensitive *M. tuberculosis* H37Rv strain with MICs of LysB and rifampicin lowered from 0.20 and 0.06 µg/mL alone to 0.10 and 0.007 µg/mL in combination, respectively, with fractional inhibitory concentration index (FICI) of ≤0.62 (Fig. 5A and B). Similar results were obtained for drug-resistant *M. tuberculosis* MDR isolate with MICs of LysB and rifampicin lowered from 0.75 and >20 µg/mL alone to 0.37 and 10 µg/mL in combination, respectively, with FICI of ≤1.0 (Fig. 5C and D). Checkerboard experiments were repeated twice to confirm the results, and results were consistent.

**TABLE 3:** Checkerboard assay of LysB in combination with rifampicin against drug-sensitive *M. tuberculosis* H37Rv strain and MDR *M. tuberculosis* isolate*,* respectively^*a*^

Drug combination	MIC (µg/mL)		FIC	FICI
	Alone	Combination		
***M. tuberculosis*** **H37Rv strain**				
LysB	0.20	0.10	0.5	0.62
Rifampicin	0.06	0.007	0.12	
**MDR** ***M. tuberculosis*** **isolate**				
LysB	0.75	0.37	0.5	1.0
Rifampicin	>20	10	0.5	

^*a*^ Synergy is defined as fractional inhibitory concentration index (FICI) FICI of ≤0.5, 0.5 < FICI ≤ 1.0 as additive, 1.0 < FICI ≤ 4.0 as indifferent, and FICI >4.0 as antagonism.

### Intracellular killing activity of LysB

**FIG 6:**
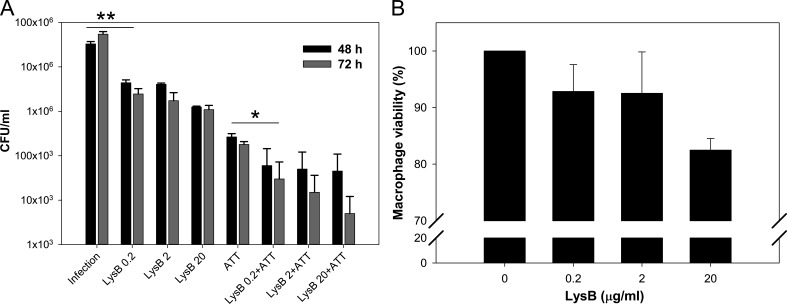
Killing of intracellular *M. tuberculosis* by D29 LysB. (**A**) RAW 264.7 macrophages infected with *M. tuberculosis* (multiplicity of infection [MOI], 10) for 3 h were treated with 0.2, 2.0, or 20.0 µg/mL of LysB either alone or in combination with ATTs, i.e., isoniazid (3 µg/mL) and rifampicin (3 µg/mL) for 48 or 72 h, respectively. The differences in the intracellular survival of *M. tuberculosis* between different treatments were analyzed by the Student’s *t*-test. Each bar represents the average value of three independent experiments, and the error bars represent the standard deviations. Significant differences are apparent between groups in pairwise comparisons using a two-tailed *t*-test at a confidence interval of *P* < 0.1 (*) or *P* < 0.05 (**). (**B**) *In vitro* cytotoxicity study of RAW 264.7 macrophage cells after 24 h of exposure to LysB. Assays were carried out in triplicate; error bars represent standard deviations. ATT, anti-TB drug.

*M. tuberculosis* is an intracellular pathogen which resides inside macrophages, and the anti-TB drugs are able to target these bacilli. However, in order to use LysB as an effective anti-TB therapeutic, it is important to examine if it can target *M. tuberculosis* residing inside macrophages. Thus, to check the ability of LysB to kill intracellular *M. tuberculosis*, the RAW 264.7 macrophages were infected with *M. tuberculosis* H37Rv. Following infection for 3 h, to investigate the effect of time and increasing concentration of LysB, infected cell lines were treated with 0.2, 2.0, or 20.0 µg/mL of LysB for 48 or 72 h. Our data clearly show the dose and time-dependent mycobacterial killing by LysB (Fig. 6A). A significant difference was observed between the infected and non-treated and infected and treated groups. The observed activity was comparable to that elicited by ATT (isoniazid and rifampicin). To evaluate whether combination therapy of LysB and ATT would enhance the efficacy, we treated macrophages with ATT along with different concentrations of LysB. Interestingly, benefits of combined LysB–antibiotic therapy for the control of mycobacteria under *in vitro* condition were also visualized against intracellular *M. tuberculosis*. The combination of ATT and LysB (concentration: 3 µg/mL each of isoniazid (INH) and rifampicin (RIF) and 0.2 µg/mL, respectively) significantly reduced the mycobacterial survival compared to the individual treatment (*P* < 0.1) (Fig. 6A). These results suggest that LysB can synergize with anti-TB drugs and potentiate its action against intracellular *M. tuberculosis*. The results also suggest the compatibility of LysB with two main anti-TB drugs, i.e., isoniazid and rifampicin.

We also evaluated the cyto-compatibility of LysB with macrophages using (3-[4,5-dimethylthiazol-2-yl]-2,5-diphenyltetrazolium bromide) (MTT) assay. RAW 264.7 mouse macrophages were incubated with various concentrations of LysB for 24 h, and cells were assessed based on the activity of the live cells to convert tetrazolium MTT to purple formazan. Treatment with 1% solution of Triton X-100 served as positive control and resulted in complete cell lysis. Incubation with LysB at all the tested concentrations showed no significant change in macrophage viability, thus demonstrating no signs of cytotoxicity (Fig. 5B). These results indicate that LysB is cyto-compatible with macrophages.

## DISCUSSION

Tuberculosis mostly occurs as a chronic infection and is one of the major global health threats. The emergence, evolution, and spread of drug-resistant strains have put pressure to update the chemotherapy regimen for faster and efficient treatment. The presence of unique cell wall has made it difficult for the drug to go inside and act on the pathogen. Thus, there is an urgent need to search for new sources or agents having either anti-mycobacterial activities or an ability to potentiate the existing regimen.

Mycobacteriophage is a bacterial virus that has the ability to infect and kill mycobacteria. Mycobacteriophage D29 is able to infect and kill *M. tuberculosis*, and hence, it has been considered as potential next-generation therapeutics (15, 27). D29 phage encodes for LysA, LysB, and holin to target peptidoglycan cell wall, mycolic acid layer, and the cell membrane, respectively, in order to release phage progeny at the end of the lytic cycle (21). D29 LysB protein possesses esterase activity that can break down mycolylarabinogalactan present in the mycobacterial cell wall (17). One study evaluated activity of D29 mycobacteriophage-derived endolysins against mycolylarabinogalactan–peptidoglycan isolated from *M. smegmatis*, while another study evaluated the effect of MS6 LysB activity against extractable lipids from *M. smegmatis*, *Mycobacterium bovis* BCG, and *M. tuberculosis* H37Ra (11, 17). However, its activity against viable planktonic and intracellular *M. tuberculosis* and against *M. tuberculosis* MDR isolates has not yet been reported. Further, no studies have looked into the effect of inoculum size on LysB activity. We therefore, investigated the activity of D29-derived LysB against TB-causing bacteria.

In this study, we used two different lytic assay procedures to quantify the activity of D29 LysB against *M. tuberculosis*. Mycobacterial survival quantified by REMA assay suggested that the addition of LysB even at a very low concentration inhibited *M. tuberculosis* growth. The MIC of LysB against MDR clinical isolate was found to be 0.75 µg/mL. Our observations were similar to the study by Fraga et al., who reported the MIC of LysB for *M. tuberculosis* and *Mycobacterium ulcerans* (20). The observed MIC was strain dependent and was found to be 0.20 and 0.079 µg/mL, respectively, for *M. tuberculosis* and *M. ulcerans.* It has been suggested that the observed strain-dependent variation in MIC can be attributed to an exceptionally complex cell wall based on sugars and lipids, which differs between pathogenic and non-pathogenic mycobacteria and also accounts for difference in pathogenesis (28). The ultra-structural study by Singh et al. has also revealed that there is thickening of the cell envelope in drug-resistant isolates and has been assumed to be one of the primary reasons for observed resistance toward rifampicin and isoniazid (29).

To further characterize the activity levels, plate lysis assays were performed with LysB. Plate lysis assay used a high-level inoculum compared with the REMA assay. The results in the plate lysis assay with *M. tuberculosis* were generally similar to the REMA assay, though there was a shift in the lowest concentration of LysB at which activity was observed (0.20 µg/mL to 3 µg/mL). In order to confirm whether the observed shift was due to inoculum size (10^5^ CFU/mL in REMA assay to 10^7^ CFU/mL in plate lysis assay), we further investigated the effect of inoculum size on drug activity against *M. tuberculosis* by broth micro-dilution method. In our study, the increase in inoculum size from 10^5^ to 10^6^ CFU/mL in REMA assay resulted in shift in rifampicin MIC from 0.02 to >5.0 µg/mL. Several studies have shown that the efficacy of anti-microbials is influenced by inoculum size. Taneja and Tyagi found MIC higher than reported MICs for anti-TB drugs at higher inoculums (22). MICs were similar to the reported values with the inoculum size of 5 × 10^4^ CFU/well as was used in the present study. A study by Banfi et al. noted a significant change in MIC of streptomycin, i.e., from 0.25 to 1.0 µg/mL against *M. tuberculosis* following a 10-fold increase in inoculum concentration. The same study reported a twofold increase in rifampicin MIC following a 10-fold shift in mycobacterial concentration (30). A study by Jung et al. also confirmed that inoculum size affects drug susceptibility test against mycobacteria, leading to reduced drug activity from the increased bacterial cell density (30).

Tween-80 is commonly used during mycobacterial culture in order to reduce the aggregates due to bacterial surface hydrophobicity. It has been suggested that Tween-80 may promote the anti-mycobacterial activity of LysB due to the action of the oleic acid released by hydrolysis of the surfactant by LysB or due to the interaction of Tween-80 with mycobacterial cell wall component inducing membrane permeabilization (11, 31). In our study, LysB exhibited significant anti-bacterial activity in both the presence and the absence of Tween-80. The observed results are supported by the additive effect with rifampicin, an anti-mycobacterial drug, which probably resulted from increased cell wall permeability in the presence of LysB. A separate study showed the activity of D29 LysB against *M. tuberculosis* mc^2^7902_Lux, and the observed MIC in the presence or absence of Tween-80 was 0.08 or ≥0.4 µM, respectively (19). A study showed that D29 LysB demonstrated equal activity against both Tween-80 and Tween-20 (12). As MIC of D29 LysB was lower in the presence of Tween-80, keeping in view of future translation into development of product for clinical use where Tween-80 has been successfully included in inhaled therapeutics, we tested LysB activity in the presence or absence of Tween-80.

The therapeutic efficacy of LysB treatment depends on the presence of biologically active LysB *in vivo*. Studies have shown a rapid decrease in endolysin levels after administration, leading to loss in activity over time (32, 33). Based on these observations, we decided to investigate the effect of LysB on survival of intracellular mycobacteria in *ex vivo* macrophage infection model. In our study, LysB administration effectively decreased the bacterial load in the infected macrophages by almost 50% at the MIC dose of 0.20 µg/mL. Our observations are similar to the recent work by various laboratories who reported the activity of endolysins against intracellular bacteria. Lai et al. reported that the treatment of *M. smegmatis* infected RAW 264.7 macrophages with BTCU-1-derived LysB (5 µg/mL) for 12 h reduced the survival of bacteria by 80% following treatment (13); it may be noted here that LysB of BTCU-1 shows 63% sequence similarity with that from D29 LysB. Another study by Shen et al. showed that bacteriophage-encoded PlyC endolysin can cross epithelial cell membranes and clear intracellular *Streptococcus pyogenes* in a dose-dependent manner (34). The present study is limited by lack of mechanistic study facilitating internalization of LysB within the intracellular environment. Moreover, before using LysB as a potential therapeutic for TB treatment, a number of important factors such as drug toxicity, immunogenicity, efficacy, resistance, and synergy are to be investigated. A number of pre-clinical studies suggest that lysin administration by topical, systemic, or intravenous route has no harmful, abnormal, or irritant side effects (35 – 37). Several studies have shown that though lysins can elicit an immune response, this does not neutralize their activity or prevent their use as anti-bacterial in the treatment of systemic infections (38, 39). Nevertheless, data on the pharmacokinetics and pharmacodynamics of endolysins as potential drugs are limited. A study showed no signs of toxicity in the rodent single- or repeated-dose tests for central nervous system function of respiratory function. Although multiple dosing led to some adverse effect, it was noted to be mild and self-limiting (36). Although the results of safety evaluations and immunogenicity studies to date largely support the idea of endolysins as a potential therapeutic, a greater volume of research in this area needs to be carried out to ensure that different classes of lysins do not pose a significant risk to the host. Further, the occurrence of lysin-resistant bacteria is unlikely since phages have naturally evolved with their bacterial hosts over millions of years to produce these enzymes that are essential for the release of their progeny. Nevertheless, studies with different clinical isolates of both drug-sensitive and drug-resistant *M. tuberculosis* are required to look into the effect of variability of TDM on LysB efficacy.

Nonetheless, our data indicate that the D29 LysB can be internalized within macrophages and that the protein retains bacteriolytic efficacy against *M. tuberculosis*. For therapeutic use, an anti-microbial agent should not affect mammalian cells and only target the pathogen. In our study, the treatment of RAW 264.7 macrophages with LysB even at the highest tested concentration, i.e., 20 µg/mL, did not result in a significant decrease in viability, and the observed effect was due to LysB activity within the intracellular environment. The findings were similar to those of Shen et al., where treatment of eukaryotic cells with PlyC lysin did not affect the membrane integrity (34). The study by van Schie et al. also discussed that lung delivery of therapeutic enzyme up to 0.13 mg/mL is possible without adverse effect (19). The observed MIC in our study is much lower than estimated doses, and it may be decreased further in the presence of Tween-80, which has been successfully included in inhalation therapy. The observed synergy between anti-TB drugs and LysB against drug-susceptible and MDR *M. tuberculosis* isolates in the present study suggests that D29 LysB when combined with anti-TB drugs can improve the therapeutic efficacy of anti-TB drugs even against drug-resistant tuberculosis and may even shorten the duration of treatment.

### Conclusions

The present report is the first of its kind demonstrating the effect of LysB against *M. tuberculosis* drug-susceptible and MDR isolates both in intracellular and extracellular environments. The study also showed that although Tween-80 potentiated the activity of LysB, it is not essential for its activity. Importantly, LysB could be used as an adjuvant to improve the current antibiotic regimen, given its *in vitro* additive effect when it is provided in combination with isoniazid and rifampicin. Although the present study did not evaluate the efficacy of LysB in the animal model, nevertheless, we present a proof of concept of the anti-microbial activity of LysB against *M. tuberculosis*. Systematic research is required to explore the activities of D29 LysB both in pre-clinical and clinical trials before they may be used in adjunctive therapy with standard anti-TB drugs for better management of the disease. Special attention should be given to the studies on safety parameters before incorporation in the present treatment regimen to improve the existing therapeutic practices. Nonetheless, our study lays the foundation for an alternative treatment regimen by providing proof of concept of the anti-microbial activity of LysB against *M. tuberculosis* infection.

## MATERIALS AND METHODS

### Bacteria and culture conditions

*M. tuberculosis* H37Rv and characterized *M. tuberculosis* MDR isolate resistant to rifampicin and isoniazid (JAL-19187 obtained from the repository of National JALMA ICMR, Agra, India) were used for the study. *M. tuberculosis* strains were grown in Middlebrook 7H9 broth (Difco, USA) supplemented with ADC (Himedia, India), at 37°C with shaking, or on Middlebrook 7H11 agar (Difco) supplemented with 0.5% glycerol and 10% OADC (BD Biosciences, USA) for plate lysis assay.

### Cloning, protein expression, and lyophilization of LysB

The *gp12* gene coding for LysB was PCR amplified using a set of primers (gp12_for: 5′ CCTGGAACCGGCTAGCAAGCCCTGGCTGTTCACC and gp12_rev: 5′ CACCCCGCCTCGCGGCCGCGATCTGTCGTAGGAACTCG) and D29 genomic DNA as template, and cloning was carried out in pET21b between NheI and NotI sites to yield a hexa-histidine tag at the C-terminus of the expressed protein. LysB was purified on Ni-NTA affinity chromatography as described elsewhere with some modifications (21). Briefly, *E. coli* BL21(DE3) carrying the desired plasmid was grown at 37°C with constant shaking at 200 rpm until the OD_600_ reached 0.6. The culture was then induced by addition of Isopropyl-β-D-1-thiogalactopyranoside (IPTG) to a final concentration of 0.5 mM and was further grown for 12 h at 22°C with constant shaking at 150 rpm. The cells were harvested by centrifugation and lysed by sonication after resuspending in lysis buffer (40 mM Tris–Cl, pH 8.0, 400 mM NaCl, 5 mM imidazole, and 5 mM 2-mercaptoethanol). The lysate was clarified by centrifugation, and the supernatant was incubated with Ni-NTA (Qiagen) beads pre-equilibrated with lysis buffer for 2 h. Beads were washed by 25 column volumes of washing buffer (40 mM Tris–Cl, pH 8.0, 400 mM NaCl, 25 mM imidazole, and 5 mM 2-mercaptoethanol). The protein was then eluted in elution buffer (40 mM Tris–Cl, pH 8.0, 400 mM NaCl, 300 mM imidazole, and 5 mM 2-mercaptoethanol). The eluted protein was examined on SDS-PAGE. Fractions containing the highest amount of protein were dialyzed twice against the storage buffer (40 mM Tris–Cl, pH 8.0, 200 mM NaCl, 1 mM dithiothreitol (DTT)). This was followed by dialysis against water to remove salts. After three to four successive changes, the insoluble protein was separated by centrifugation, and the clarified solution was dialyzed against deionized water and lyophilized. The lyophilized protein was stored at –20°C or room temperature as needed. Before use, 5 mg of lyophilized protein was suspended in phosphate-buffered saline (PBS) and quantified for LysB protein using BCA kit (Sigma Aldrich, USA) following manufacturer’s protocol.

### Activity against p-nitrophenyl esters

*In vitro* esterase activity of D29 LysB was determined by treating 10 mM of p-nitrophenyl ester (Sigma) in 25 mM Tris–Cl buffer (pH 8.0) with different amounts of LysB (1.0, 2.5, 5.0, and 10.0 µg/mL); BSA (25 µg/mL) was used as negative control. The reaction was monitored by measuring the absorbance at 415 nm for 5 min at 37°C.

### Determination of *M. tuberculosis* susceptibility to D29 mycobacteriophage LysB

The MIC determination of D29 LysB against *M. tuberculosis* and *M. tuberculosis* MDR isolate was performed using the REMA method (40). Briefly, D29 LysB (0.03–12.0 µg/mL for drug-sensitive *M. tuberculosis* H37Rv strain and 0.05–100.0 µg/mL for *M. tuberculosis* MDR isolate) suspended in protein buffer (40 mM Tris–Cl, pH 8.0, 200 mM NaCl, and 1 mM DTT) was diluted serially twofold in Middlebrook 7H9 containing ADC (10%), and 100-µL of it was placed in a 96-well microtiter plate. The medium used during the experiments was supplemented with Tween-80 (0.05%). *M. tuberculosis* grown in Middlebrook 7H9 medium was harvested at 2,500 × *g*, and bacterial concentration was adjusted to 1.0 McFarland standard. To determine the effect of inoculum size, the phage lysin activity was determined at two different bacterial concentrations, i.e., 0.05 McFarland unit (equivalent to ~1 × 10^5^ CFU/mL) (recommended for determination of antibiotic susceptibility by REMA method) (22) or 1 McFarland standard (equivalent to ~2 × 10^6^ CFU/mL) (41). Rifampicin was used as the positive control. For the *M. tuberculosis* MDR isolate, LysB MIC determination was done at bacterial concentration of 1 × 10^5^ CFU/mL. Sterile distilled water (200 µL) was distributed in the perimeter wells of the microtiter plate to avoid dehydration of medium during incubation (Himedia; 96 well plate, flat bottoms).

The plate seeded with 1 McFarland unit CFU was incubated at 37°C for 48 h, while the plate seeded with 0.05 McFarland unit CFU was incubated for 6 days. After incubation for the respective time period at 37°C, resazurin dye (0.02%) (Sigma Aldrich, Germany) was added to all the wells and incubated overnight before visualization of change in color from blue to pink. Visual MIC was defined as the lowest concentration of drug that prevented a color change. The assays were performed in entirety with two replicates on at least two separate occasions. To confirm the results visualized by REMA assay, each well was mixed using a pipette and 3 µL aliquots were spotted onto Middlebrook 7H11 agar plates supplemented with OADC and incubated for 3–4  weeks at 37°C (42).

### Screening of D29 LysB against *M. tuberculosis* by plate lysis method

To test the activity of LysB against *M. tuberculosis*, purified proteins (final concentration 1.5–400.0 μg/mL) were spotted onto a freshly spread lawn of log-phase *M. tuberculosis*. The double agar overlay technique was used to identify the anti-mycobacterial activity of lysin. Briefly, soft agar was prepared by adding agar (0.8%) to 7H9 media along with the required supplements. The suspension was autoclaved and allowed to cool down to 42°C. Late log phase bacterial culture (0.5 mL) having OD_600_ ≤1 (~1 × 10^7^ CFU/mL) was added to 4.5 mL of the soft agar and was poured onto an MB 7H11 agar plate supplemented with 10% OADC (oleic acid, albumin, dextrose, and catalase). In a parallel experiment, the soft agar was supplemented with Tween-80 (1%) to evaluate the effect of Tween-80 on LysB activity. The soft agar was allowed to cool down and solidify for 45 min-1 h. The 10 µL solution of purified LysB were spotted onto bacterial lawns and was air dried for 30 min. Protein buffer was spotted as a negative control. Plates were incubated at 37°C for approximately 2–3 weeks until visible lawns were obtained. Anti-microbial activity of LysB was indicated by a clear lysis zone within the lawn wherever *M. tuberculosis* growth was inhibited.

### Determination of D29 LysB interactions with anti-TB drugs using a REMA checkerboard assay

To determine whether LysB interact synergistically, additively or antagonistically, checkerboard assay experiments were performed in a 96-well plate against drug-sensitive *M. tuberculosis* H37Rv and multi-drug-resistant *M. tuberculosis* MDR isolate, respectively (Fig. 5A through D). For *M. tuberculosis* MDR isolate, in the first step, seven concentrations of D29 LysB (ranging from 0.09 to 6.0 µg/mL) were double diluted column-wise, while six concentrations of rifampicin (ranging from 0.16 to 10.0 µg/mL) were double diluted row-wise. For drug-sensitive *M. tuberculosis* H37Rv strain, D29 LysB concentration ranged from 0.05 to 3.2 µg/mL while rifampicin concentration ranged from 0.003 to 0.25 µg/mL, respectively. Mycobacterial culture (100 µL) containing 1 × 10^5^ CFU/mL was added to each well and incubated for six days. Resazurin dye was added, and results were recorded after overnight incubation. The results were interpreted as the FICI calculated as FIC of D29 LysB + FIC of drug. FIC was calculated as MIC of LysB or drug in combination/MIC of LysB or drug alone. FICI values of ≤0.5, >0.5–1.0, >1.0–4.0, and >4.0 were interpreted as indicating “synergy,” “additive,” “indifference,” and “antagonism,” respectively (43).

### Intracellular bactericidal assay

Mouse peritoneal macrophage cell line RAW 264.7 (procured from National Centre for Cell Sciences, Pune, India) was used to study the treatment of intracellular mycobacterial infection as previously standardized in our laboratory (44). RAW 264.7 macrophages were cultured in Dulbecco’s Modified Eagle Medium (Himedia) supplemented with 10% heat in-activated fetal bovine serum (Himedia) and L-glutamine (2 mM). RAW 264.7 macrophages (2 × 10^5^ cells/well) were seeded into a 96-well tissue culture plate (Himedia) and incubated overnight at 37°C with an atmosphere of 5% CO_2_. *M. tuberculosis* H37Rv was used to infect RAW 264.7 macrophages in the ratio of 10:1. Following infection with *M. tuberculosis* H37Rv for 3 h, the supernatant was removed, and wells were washed thrice with 1× PBS. The infected macrophage monolayers were subsequently treated for 48 or 72 h with either 1×, 10×, or 100× MIC of LysB. The control group included macrophages treated with either anti-TB drugs, i.e., isoniazid and rifampicin (3 µg/mL each), or cells without treatment (negative control) (45). Following incubations at respective time points, cell monolayers were washed three times and lysed with 140 µL of 0.01% Triton X-100. The cell lysates were plated on Middlebrook 7H11 agar to quantify viable intracellular *M. tuberculosis.* The test was repeated thrice and the number of CFUs recovered per well (mean number ±S.D.) was determined.

### Cell viability assay

The cell viability of RAW 264.7 macrophages following treatment with D29 LysB was evaluated by MTT assay as described previously (44). Briefly, RAW 264.7 macrophages (2 × 10^5^ cells/well) were seeded as described before. The adhered cells were treated with LysB at concentrations of 0.2, 2.0, and 20.0 µg/mL (equivalent to 1×, 10×, or 100× MIC of LysB) for 24 h. Blank well containing medium only and untreated wells were included as controls. At specified time points, MTT reagent (Himedia) to an amount equal to 10% of the total volume of wells was added and incubated for 3–4 h in the dark. Formazan crystals formed were dissolved in 100 µL of dimethyl sulfoxide and mixed by pipetting up and down, and the resultant absorbance was measured at 570 nm using a microplate spectrophotometer (BioTek Epoch, USA).

### Statistical analysis

The data are expressed as the mean ± standard error of the mean. CFUs were enumerated, and the data were analyzed between groups in pair-wise comparisons using a two-tailed *t*-test. *P* value of <0.05 was considered statistically significant. OriginLab software (version 6.0; Origin Lab Corp., Northampton, MA, USA) was used for all the calculations.
